# Associations of Metabolic Phenotypes with Cardiac Structure and Clinical Outcomes: Insights from the UK Biobank

**DOI:** 10.1210/clinem/dgaf684

**Published:** 2025-12-22

**Authors:** Balázs Bogner, Matthias Jung, Marco Reisert, Juliane Maushagen, Susanne Rospleszcz, Janis M Nolde, Christopher L Schlett, Fabian Bamberg, Andreas Kammerlander, Jakob Weiss, Jana Taron

**Affiliations:** Department of Diagnostic and Interventional Radiology, University Medical Center Freiburg, Faculty of Medicine, University of Freiburg, Freiburg 79106, Germany; Berta-Ottenstein-Programme, Faculty of Medicine, University of Freiburg, Freiburg 79106, Germany; Department of Diagnostic and Interventional Radiology, University Medical Center Freiburg, Faculty of Medicine, University of Freiburg, Freiburg 79106, Germany; Division of Medical Physics, Department of Diagnostic and Interventional Radiology, University Medical Center Freiburg, Faculty of Medicine, University of Freiburg, Freiburg 79106, Germany; Department of Stereotactic and Functional Neurosurgery, University Medical Center Freiburg, Faculty of Medicine, University of Freiburg, Freiburg 79106, Germany; Department of Diagnostic and Interventional Radiology, University Medical Center Freiburg, Faculty of Medicine, University of Freiburg, Freiburg 79106, Germany; Department of Diagnostic and Interventional Radiology, University Medical Center Freiburg, Faculty of Medicine, University of Freiburg, Freiburg 79106, Germany; Department of Internal Medicine IV (Nephrology), University Medical Center Freiburg, Faculty of Medicine, University of Freiburg, Freiburg 79106, Germany; Department of Diagnostic and Interventional Radiology, University Medical Center Freiburg, Faculty of Medicine, University of Freiburg, Freiburg 79106, Germany; Department of Diagnostic and Interventional Radiology, University Medical Center Freiburg, Faculty of Medicine, University of Freiburg, Freiburg 79106, Germany; Department of Cardiology, Medical University of Vienna, Vienna General Hospital, Vienna 1090, Austria; Department of Diagnostic and Interventional Radiology, University Medical Center Freiburg, Faculty of Medicine, University of Freiburg, Freiburg 79106, Germany; Department of Diagnostic and Interventional Radiology, University Medical Center Freiburg, Faculty of Medicine, University of Freiburg, Freiburg 79106, Germany

**Keywords:** cardiovascular diseases, magnetic resonance imaging, metabolism, mortality, obesity

## Abstract

**Context:**

Metabolic syndrome and obesity represent major cardiovascular risk factors, yet their combined effects on cardiac structure and clinical outcomes remain incompletely understood.

**Objective:**

To examine associations between metabolic phenotypes, cardiac magnetic resonance imaging (CMR), and clinical outcomes.

**Design:**

Prospective study with a median 5.1-year follow-up.

**Setting:**

Population-based cohort from the UK Biobank.

**Participants:**

A total of 22 789 participants (mean age 64.1 ± 7.5 years, 52.6% female) were categorized as metabolically healthy nonobese (MHN) vs obese (MHO) or unhealthy nonobese (MUN) vs obese (MUO), based on obesity (body mass index ≥30 kg/m²) and metabolic status (prevalent diabetes or both hypertension and hyperlipidemia).

**Main Outcome Measure(s):**

Primary and secondary endpoints were major adverse cardiovascular events (MACE) and all-cause mortality, respectively. CMR included left ventricular ejection fraction (LVEF, %), end-diastolic volume (LVEDV, mL), myocardial mass (g), wall thickness (mm), cardiac output (L/min), and left atrial volume (mL). Linear regression and Cox proportional hazards models, adjusted for age, sex, and smoking, assessed associations between metabolic phenotypes, CMR, and clinical outcomes.

**Results:**

In adjusted models, all phenotypes demonstrated increased WT (MHO: β=.53 [.50, .55]; MUN: β=.24 [.21, .27]; MUO: β=.66 [.61, .70]), compared to MHN. LVEDV was increased in obesity phenotypes (MHO: β=4.67 [4.07, 5.27]; MUO: β=5.14 [4.05, 6.22]) and decreased in MUN (β=−1.25 [−1.95, −.55]), while MUO showed reduced LVEF (β=−.48 [−.91, −.04]). MACE risk was increased in unhealthy phenotypes (MUN: adjusted hazard ratio (aHR) = 1.55 [1.16-2.07]; MUO: aHR = 1.95 [1.28-2.97]). All phenotypes showed increased all-cause mortality risk (MHO: aHR = 1.65 [1.23-2.21]; MUN: aHR = 1.41 [1.05-1.90]; MUO: 2.10 [1.41-3.15]).

**Conclusion:**

Metabolic phenotypes show distinct cardiac structural changes and increased mortality risk, supporting their potential in cardiovascular risk stratification.

The global rise in obesity represents a major public health challenge (1, 2). According to the World Health Organization, over 1 billion people are living with obesity worldwide, accounting for about 2.8 million deaths annually (3). Obesity is often accompanied by metabolic syndrome (MetS), thus contributing to an increased risk of cardiovascular diseases (CVD) (4, 5). Precise cardiovascular risk assessment is critical to reduce mortality and preserve quality of life in these patients. However, obesity classification remains challenging due to the presence of distinct obesity/metabolic phenotypes, each associated with unique cardiovascular risk profiles (6). Based on (1) MetS components, including arterial hypertension, diabetes, hyperlipidemia, and (2) a body mass index (BMI) < 30 or ≥30 kg/m^2^, 4 metabolic phenotypes have been identified (7): metabolically healthy nonobese (MHN), metabolically healthy obese (MHO), metabolically unhealthy nonobese (MUN), and metabolically unhealthy obese (MUO). As obesity alone may not reflect the metabolic health state, MHO was introduced to characterize individuals with obesity but without manifest metabolic alterations (8). Conflicting data exist on whether MHO is linked to elevated risk of CVD and major adverse cardiovascular events (MACE) (9-11).

Obesity and MetS affect the heart through multiple mechanisms, including hemodynamic alterations, metabolic dysfunction, and neurohormonal activation (12). The resulting changes in cardiac structure and function may contribute to increased CVD prevalence (13). Despite this established mechanistic link, comprehensive studies investigating cardiac adaptation patterns across metabolic phenotypes are lacking.

The UK Biobank (UKB) is a prospective population-based cohort study, which offers a unique opportunity to examine the relationship between metabolic phenotypes, cardiac function, and CVD outcomes in a large sample of the general population as it provides detailed demographic and clinical data along with cardiac magnetic resonance imaging (CMR) data, the gold standard for cardiac function assessment (14).

In this study, we investigated the association between metabolic phenotypes, CMR-derived cardiac structure, function, and clinical outcomes (MACE and all-cause mortality) in the UKB. We hypothesized that metabolic phenotypes are characterized by distinct CVD risk profiles with corresponding changes in cardiac structure and function and are associated with MACE and all-cause mortality beyond standard clinical risk factors.

## Materials and Methods

### Ethical Approval

This study was conducted under the UKB's general ethical approval from the NHS National Research Ethics Service. All UKB participants provided written informed consent at enrollment.

### Study Design and Participants

**Figure 1.**
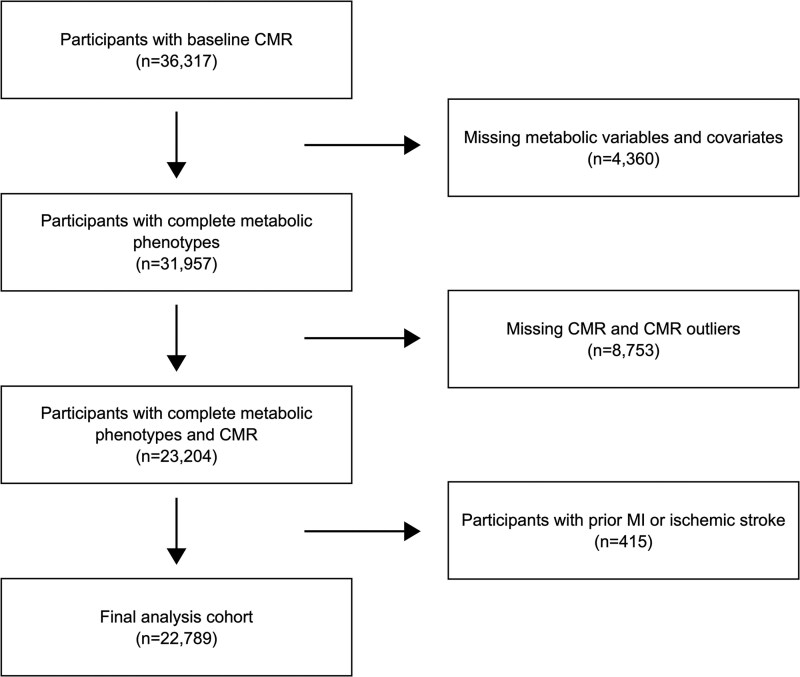
Consort diagram.

This study is based on the UKB, a large prospective cohort study of approximately 500 000 participants aged 40 to 69 years between 2006 and 2010 (15). The baseline assessment involved the collection of extensive questionnaire data, physical measurements, and biological samples. Health outcomes are prospectively tracked through linkages with national electronic health record systems and recontact with participants. In 2014, the UKB initiated an imaging substudy of a randomly selected 20% (n = 100 000) subset of the original participants. The imaging protocol consists of multiorgan multimodality imaging, including CMR (16). The current analysis included UKB participants (n = 36 317) who underwent CMR between April 30, 2014, and September 20, 2021. The median time between baseline assessment and CMR was 9.0 years [interquartile range (IQR): 7.5-10.1; range: 3.8-14.1 years]. We excluded participants with (1) missing metabolic variables or covariates (n = 4360), (2) missing or outlier CMR parameters (n = 8753), and (3) a history of myocardial infarction [identified through International Classification of Diseases, Tenth Revision (ICD-10) codes I21-I22; International Classification of Diseases, Ninth Revision (ICD-9) codes 410-411] or ischemic stroke (identified through ICD-10 code I63; ICD-9 codes 433-434) (n = 415) prior to imaging. The final analysis cohort included 22 789 participants (Fig. 1).

### CMR Parameters

CMR scans were performed using a 1.5 Tesla scanner (MAGNETOM Aera, Syngo Platform VD13A, Siemens Healthcare, Erlangen, Germany), following the standardized UKB protocol.

The details, rationale, and challenges of the CMR imaging protocol are described in dedicated publications (17, 18). CMR-derived parameters were categorized into 2 domains: (1) left ventricular function and chamber volumes, including left ventricular ejection fraction (LVEF, %), cardiac output (CO, L/min), and left ventricular end-diastolic volume (LVEDV, mL/m) and (2) cardiac remodeling parameters including left ventricular myocardial mass (LVM, g), global left ventricular mean wall thickness (WT, mm), left atrial volume (LAV, mL), and the left ventricular myocardial mass to left ventricular end-diastolic volume ratio (LVM/LVEDV, g/mL). Heart rate was routinely monitored during CMR acquisition. To account for body size, LVEDV and CO were indexed to height (reported as mL/m and L/min/m, respectively), while LVM and LAV were indexed to body surface area (BSA; reported as g/m² and mL/m², respectively). BSA was calculated using the Mosteller formula (19):


$$BSA\;(m^{2})=\frac{\sqrt{\mathrm{height}\;(\mathrm{cm})\;\times \;\mathrm{weight}\;(\mathrm{kg})}}{3600}$$

### Obesity

Height and weight were recorded during the CMR visit using a Seca 202 stadiometer (Hamburg, Germany) and a Tanita BC-418 body composition analyzer (Tokyo, Japan), respectively. BMI was calculated as weight (kg) divided by height squared (m²). Following World Health Organization criteria, participants with a BMI ≥ 30 kg/m² were categorized as obese (20).

### Metabolic Health

Metabolic health was determined using MetS criteria, consisting of hyperlipidemia, hypertension, and diabetes, as suggested before (7). Hyperlipidemia was defined by elevated triglycerides (≥150 mg/dL) or cholesterol levels [total cholesterol ≥5.18 mmol/L, low-density lipoprotein ≥3.37 mmol/L, or high-density lipoprotein (HDL) <1.04 mmol/L in men and <1.30 mmol/L in women], based on blood samples collected at baseline assessment. Prevalent hypertension was identified using ICD-10 codes I10-15 or ICD-9 codes 401-405, while prevalent diabetes was identified through ICD-10 codes E10-14 or ICD-9 code 250. Both hypertension and diabetes status were determined from records prior to the imaging date, capturing clinically diagnosed conditions present when CMR was performed.

### Definitions of Metabolic Phenotypes

Participants were categorized into distinct metabolic phenotypes based on the combination of their obesity and metabolic health status. Participants were classified as metabolically unhealthy by the presence of (1) diabetes (regardless of other risk factors) or (2) a combination of hypertension and hyperlipidemia (7). Using these criteria, participants were assigned to 1 of 4 phenotypes: (1) MHN, (2) MHO, (3) MUN, or (4) MUO. These criteria allow for the identification of clinically significant metabolic dysfunction independent of obesity status, enabling examination of MHO as a distinct phenotype. To assess the robustness of findings across different definitions of metabolic health, we conducted a sensitivity analysis where participants were classified as metabolically unhealthy if they had any single MetS component (hypertension, hyperlipidemia, or diabetes), rather than requiring diabetes or both hypertension and hyperlipidemia as described in Supplemental Methods [(21), Zenodo].

#### Endpoints

The primary endpoint of this study was MACE, defined as myocardial infarction (ICD-10 codes I21–I22; ICD-9 codes 410-411), ischemic stroke (ICD-10 code I63; ICD-9 codes 433-434), or CVD mortality (ICD-10 codes I00–I78). The secondary endpoint was all-cause mortality. The UKB regularly receives death notifications via linkage with national death registries. Dates of death were recorded from the start of the study up to the data download date, May 25, 2023. Follow-up time was defined as the period from the date of the magnetic resonance imaging scan (used as both the start and reference point for survival analysis) to the earliest of the following events: date of death, occurrence of an ICD-coded outcome, loss to follow-up, or May 25, 2023.

#### Clinical covariates

In addition to the aforementioned clinical covariates, the following a priori defined risk factors were included in multivariable analyses: age, sex, and smoking status (current or past) at the time point of imaging.

### Statistical Analysis

Baseline characteristics are presented as total numbers, percentages, means, and SDs. Baseline characteristics across all metabolic phenotypes were compared using the Kruskal-Wallis test (continuous variables) or Fisher's exact test (categorical variables). For pairwise comparisons (MHN vs MHO and MUN vs MUO), the Wilcoxon rank sum test (continuous variables) or Fisher's exact test (categorical variables) was used. After confirming normal distribution, differences in CMR parameters were assessed using 1-way ANOVA with Bonferroni-corrected post hoc comparisons. CMR measurements falling outside 3 IQRs beyond the first or third quartiles were classified as outliers and excluded from the analysis. Associations between metabolic phenotypes and CMR parameters were investigated using univariable and multivariable linear regression analyses, adjusted for age, sex, and smoking status. LVEDV and CO were indexed to patient height to account for patient body size. Associations between metabolic phenotypes, incident MACE, and all-cause mortality were evaluated using univariable and multivariable Cox proportional hazards models, adjusted for age, sex, and smoking status. Hazard ratios (HRs) with 95% confidence intervals (CIs) were calculated using MHN as the reference group. HRs with 95% CIs were calculated for each category within each metabolic phenotype. All *P*-values <.05 were considered statistically significant. All statistical analyses were performed using R statistics (R-4.3.0—R Core Team, https://www.r-project.org/).

## Results

### Study Population

The baseline characteristics of 22 789 participants (mean age 64.1 ± 7.5 years; 52.6% female) are displayed in Table 1, stratified by metabolic phenotypes. In total, 20 255 (88.9%) were metabolically healthy (MHN: n = 17 689 [77.6%]; MHO: n = 2566 [11.3%]), and 2534 (11.1%) were metabolically unhealthy (MUN: n = 1827 [8.0%]; MUO: n = 707 [3.1%]). Among metabolically healthy individuals, those with obesity were slightly younger (62.4 ± 7.2 vs 63.8 ± 7.6 years; *P* < .001) and had a CVD risk profile with a higher prevalence of hypertension (5.7% vs 2.8%; *P* < .001), elevated triglycerides (1.9 ± 1.1 vs 1.5 ± 0.9 mmol/L; *P* < .001), and lower HDL cholesterol (1.3 ± 0.3 vs 1.5 ± 0.4 mmol/L; *P* < .001) but had lower prevalence of smoking (former of current) (55.6% vs 61.4%; *P* < .001). We found similar patterns among metabolically unhealthy individuals. Compared to MUN, MUO were younger (66.0 ± 6.8 vs 68.0 ± 6.6 years; *P* < .001) and had higher triglyceride levels (2.2 ± 1.1 vs 2.0 ± 1.1 mmol/L; *P* < .001) and lower HDL cholesterol (1.3 ± 0.3 vs 1.4 ± 0.4 mmol/L; *P* < .001). The proportion of diabetes was higher in MUO than in MUN (27.7% vs 18.4%; *P* < .001). Participant classification using the alternative metabolic health definition resulted in substantially more participants classified as metabolically unhealthy [Table S1 (21), (Zenodo)], with corresponding baseline characteristics shown in [Table S2 (21), (Zenodo)].

**Table 1.** Baseline characteristics stratified by metabolic phenotypes

Characteristics	MHN (n = 17 689)	MHO (n = 2566)	MUN (n = 1827)	MUO (n = 707)	*P*-value All groups	*P*-value MHN vs MHO	*P*-value MUN vs MUO
Age (years)	63.8 (7.6)	62.4 (7.2)	68.0 (6.6)	66.0 (6.8)	<.001	<.001	<.001
Sex (Male/female) (%)	9488 (53.6)	1355 (52.8)	793 (43.4)	347 (49.1)	<.001	.433	.011
BMI (kg/m²)	24.4 (2.8)	33.2 (3.2)	25.6 (2.7)	33.7 (3.4)	<.001	<.001	<.001
Smoking status (current or past) (%)	10 855 (61.4)	1426 (55.6)	977 (53.5)	352 (49.8)	<.001	<.001	.101
Hypertension (%)	497 (2.8)	147 (5.7)	1661 (90.9)	644 (91.1)	<.001	<.001	.890
Total cholesterol (mmol/L)	5.7 (1.1)	5.7 (1.1)	5.8 (1.1)	5.6 (1.2)	.001	.576	.003
Triglycerides (mmol/L)	1.5 (0.9)	1.9 (1.1)	2.0 (1.1)	2.2 (1.1)	<.001	<.001	<.001
HDL cholesterol (mmol/L)	1.5 (0.4)	1.3 (0.3)	1.4 (0.4)	1.3 (0.3)	<.001	<.001	<.001
LDL cholesterol (mmol/L)	3.6 (0.8)	3.6 (0.8)	3.6 (0.8)	3.6 (0.9)	<.001	<.001	.222
HbA1c (mmol/mol)	34.4 (4.2)	35.4 (5.0)	37.5 (7.4)	39.5 (10.6)	<.001	<.001	<.001
Diabetes (%)	0 (0.0)	0 (0.0)	337 (18.4)	196 (27.7)	<.001	NA	<.001

Values are presented as mean (SD) for continuous variables and n (%) for categorical variables. *P*-values were calculated using the Kruskal-Wallis test (continuous variables) or Fisher's exact test (categorical variables) for comparing all groups and Wilcoxon rank sum test (continuous variables) or Fisher's exact test (categorical variables) for pairwise comparisons.

Abbreviations: BMI, body mass index; HbA1c, glycated hemoglobin; HDL, high-density lipoprotein; LDL, low-density lipoprotein; MHN, metabolically healthy nonobese; MHO, metabolically healthy obese; MUN, metabolically unhealthy nonobese; MUO, metabolically unhealthy obese; NA, not applicable.

#### Left ventricular function and chamber volumes

**Figure 2.**
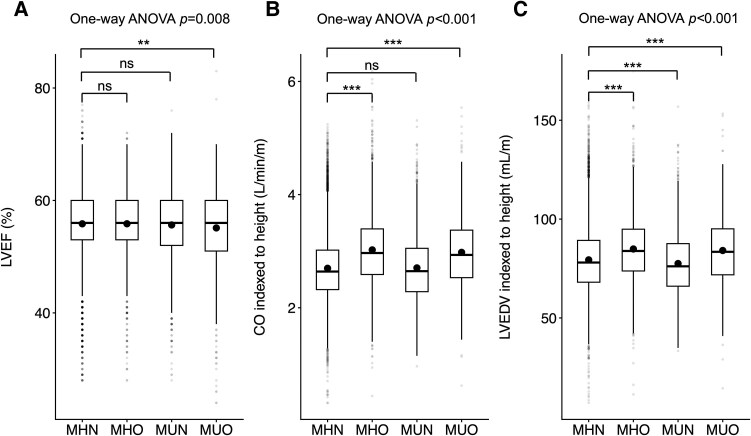
Distribution of cardiac MRI measures across metabolic phenotypes. Box plots comparing (A) LVEF (%), (B) CO indexed to height (L/min/m), and (C) LVEDV indexed to height (mL/m). In each box plot, the horizontal line represents the median, the point inside the box represents the mean, the box represents the interquartile range (25th to 75th percentiles), and the whiskers extend to 1.5 times the interquartile range. Individual data points are shown as gray dots. One-way ANOVA with Bonferroni-corrected pairwise comparisons against MHN reference group. **P < .01, ***P < .001. Abbreviations: CO, cardiac output; LVEDV, left ventricular end-diastolic volume; LVEF, left ventricular ejection fraction; MHN, metabolically healthy nonobese; ns, not significant; MHO, metabolically healthy obese; MRI, magnetic resonance imaging; MUN, metabolically unhealthy non-obese; MUO, metabolically unhealthy obese.

The MUO phenotype demonstrated significantly reduced LVEF compared to the MHN reference phenotype (55.1 ± 7.3% vs 55.8 ± 5.8%, *P* = .004, Fig. 2A). Height-indexed CO was elevated in obesity phenotypes (MHO: 3.0 ± 0.6 L/min/m, MUO: 3.0 ± 0.7 L/min/m, both *P* < .001) compared to MHN (2.7 ± 0.6 L/min/m, Fig. 2B). Height-indexed LVEDV was significantly higher in obesity phenotypes (MHO: 84.8 ± 17.1 mL/m, MUO: 84.1 ± 18.0 mL/m, both *P* < .001) but lower in MUN (77.3 ± 17.0 mL/m, *P* < .001) compared to MHN (79.3 ± 16.4 mL/m, Fig. 2C). Heart rate was higher in all phenotypes (MHO: 64.1 ± 10.1 bpm, MUN: 63.2 ± 10.5 bpm, MUO: 64.6 ± 10.9 bpm) compared to MHN (61.5 ± 9.5 bpm, all *P* < .001).

#### Cardiac remodeling parameters

LVM indexed to BSA was elevated in MUO (46.5 ± 8.7 g/m², *P* < .001) and MUN (47.1 ± 8.4 g/m², *P* < .001) compared to MHN (45.0 ± 8.1 g/m²). WT was increased across all phenotypes, with the greatest increase in obesity phenotypes (MUO: 6.3 ± 0.8 mm, MHO: 6.1 ± 0.8 mm, both *P* < .001) and a more modest increase in MUN (5.9 ± 0.7 mm, *P* < .001) compared to MHN (5.6 ± 0.7 mm). LAV indexed to BSA was significantly higher in MUO (40.2 ± 12.1 mL/m², *P* < .001) and MUN (39.1 ± 12.3 mL/m², *P* = .017) compared to MHN (38.4 ± 10.8 mL/m²). The LVM/LVEDV ratio was elevated in all 3 phenotypes (MUO: 0.7 ± 0.2 g/mL, MHO: 0.7 ± 0.2 g/mL, MUN: 0.7 ± 0.1 g/mL, all *P* < .001) compared to MHN (0.6 ± 0.1 g/mL).

### Association Between Metabolic Phenotypes and CMR Parameters

#### Left ventricular function and chamber volumes

In adjusted models, MUO was associated with reduced LVEF (β = −.48 [95% CI: −0.91, −0.04], *P* = .032), while MUN showed a positive association (β = .29 [95% CI: 0.01, 0.58], *P* = .042). Obesity phenotypes demonstrated increased height-indexed LVEDV (MHO: β = 4.67 [95% CI: 4.07, 5.27]; MUO: β = 5.14 [95% CI: 4.05, 6.22]; both *P* < .001) and CO (MHO: β = .30 [95% CI: 0.28, 0.32]; MUO: β = .31 [95% CI: 0.27, 0.35]; both *P* < .001) compared to MHN. MUN showed reduced LVEDV (β = −1.25 [95% CI: −1.95, −0.55], *P* < .001) and increased CO (β = .05 [95% CI: 0.03, 0.08], *P* < .001). Associations between metabolic phenotypes and CMR-derived functional parameters are displayed in Table 2.

**Table 2.** Associations between metabolic phenotypes and left ventricular function and chamber volumes

CMR parameters by phenotypes	Univariable model		Multivariable model	
	β (95% CI)	*P*-value	β (95% CI)	*P*-value
Left ventricular ejection fraction (%)				
MHO	.01 (−0.24, 0.25)	.954	−.04 (−0.28, 0.20)	.745
MUN	−.20 (−0.49, 0.09)	.169	.29 (0.01, 0.58)	.042
MUO	−.73 (−1.18, −.28)	.001	−.48 (−0.91, −0.04)	.032
Cardiac output indexed to height (L/min/m)				
MHO	.33 (0.30, 0.35)	<.001	.30 (0.28, 0.32)	<.001
MUN	.01 (−0.02, 0.03)	.637	.05 (0.03, 0.08)	<.001
MUO	.28 (0.24, 0.33)	<.001	.31 (0.27, 0.35)	<.001
Left ventricular end-diastolic volume indexed to height (mL/m)				
MHO	5.50 (4.82, 6.18)	<.001	4.67 (4.07, 5.27)	<.001
MUN	−1.86 (−2.65, −1.07)	<.001	−1.25 (−1.95, −0.55)	<.001
MUO	4.73 (3.50, 5.96)	<.001	5.14 (4.05, 6.22)	<.001

Beta coefficients (β) are from univariable and multivariable linear regression models, adjusted for age, sex, and smoking status. They represent the mean differences in CMR-derived cardiac parameters compared to MHN as a reference group.

Abbreviations: CI, confidence interval; CMR, cardiac magnetic resonance imaging, MHN, metabolically healthy nonobese; MHO, metabolically healthy obese; MUN, metabolically unhealthy nonobese; MUO, metabolically unhealthy obese.

#### Cardiac remodeling parameters

In adjusted models, MUO and MUN demonstrated increases in LVM indexed to BSA (MUO: β = 1.15 g/m² [95% CI: 0.67, 1.64]; MUN: β = 1.38 g/m² [95% CI: 1.07, 1.69]; both *P* < .001) compared to MHN. WT was increased across all phenotypes (MHO: β = .53 mm [95% CI: 0.50, 0.55]; MUN: β = .24 mm [95% CI: 0.21, 0.27]; MUO: β = .66 mm [95% CI: 0.61, 0.70]; all *P* < .001) compared to MHN. LAV indexed to BSA was significantly elevated in MUO (β = 1.92 mL/m² [95% CI: 1.10, 2.75], *P* < .001) and MUN (β = 1.00 mL/m² [95% CI: 0.47, 1.53], *P* < .001). The LVM/LVEDV ratio was elevated across all phenotypes (MHO: β = .06 g/mL [95% CI: 0.06, 0.07]; MUN: β = .04 g/mL [95% CI: 0.04, 0.05]; MUO: β = .08 g/mL [95% CI: 0.07, 0.09]; all *P* < .001). Associations between metabolic phenotypes and cardiac remodeling parameters are displayed in Table 3.

**Table 3.** Associations between metabolic phenotypes and cardiac remodeling parameters

CMR parameters by phenotypes	Univariable model		Multivariable model	
	β (95% CI)	*P*-value	β (95% CI)	*P*-value
Left ventricular myocardial mass indexed to BSA (g/m^2^)				
MHO	.31 (−0.03, 0.65)	.078	.11 (−0.16, 0.37)	.427
MUN	2.16 (1.77, 2.56)	<.001	1.38 (1.07, 1.69)	<.001
MUO	1.50 (0.89, 2.12)	<.001	1.15 (0.67, 1.64)	<.001
Global left ventricular mean wall thickness (mm)				
MHO	.53 (0.50, 0.56)	<.001	.53 (0.50, 0.55)	<.001
MUN	.35 (0.32, 0.39)	<.001	.24 (0.21, 0.27)	<.001
MUO	.71 (0.66, 0.77)	<.001	.66 (0.61, 0.70)	<.001
Left atrial volume indexed to BSA (mL/m^2^)				
MHO	−.16 (−0.61, 0.29)	.490	−.25 (−0.71, 0.20)	.270
MUN	.74 (0.22, 1.27)	<.001	1.0 (0.47, 1.53)	<.001
MUO	1.80 (0.98, 2.62)	<.001	1.92 (1.10, 2.75)	<.001
Left ventricular myocardial mass/left ventricular end-diastolic volume ratio (g/mL)				
MHO	.06 (0.06, 0.07)	<.001	.06 (0.06, 0.07)	<.001
MUN	.06 (0.06, 0.07)	<.001	.04 (0.04, 0.05)	<.001
MUO	.09 (0.08, 0.10)	<.001	.08 (0.07, 0.09)	<.001

Beta coefficients (β) are from univariable and multivariable linear regression models, adjusted for age, sex, and smoking status. They represent the mean differences in CMR-derived cardiac parameters compared to MHN as a reference group.

Abbreviations: CI, confidence interval, CMR, cardiac magnetic resonance imaging; MHN, metabolically healthy nonobese; MHO, metabolically healthy obese; MUN, metabolically unhealthy nonobese; MUO, metabolically unhealthy obese.

### Association Between Metabolic Phenotypes and Clinical Outcomes

**Figure 3.**
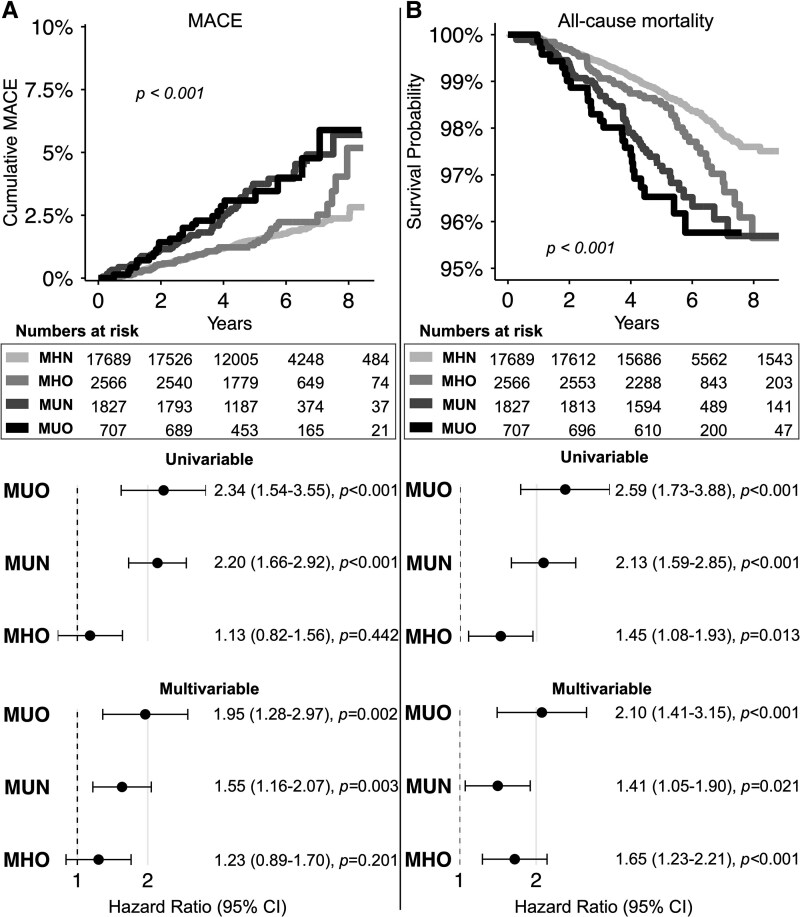
Association of metabolic phenotypes with clinical outcomes. (A) Cumulative MACE incidence curves and hazard ratios (univariable and multivariable) from Cox proportional hazards models with MHN as reference. (B) All-cause mortality Kaplan-Meier curves and hazard ratios (univariable and multivariable) from Cox proportional hazards models with MHN as reference. Numbers at risk are shown below the curves. All multivariable models were adjusted for age, sex, and smoking status. Log-rank *P* < .001 for both outcomes. Hazard ratios are shown with 95% CI. Abbreviations: CI, confidence interval; MACE, major adverse cardiovascular events; MHN, metabolically healthy nonobese; MHO, metabolically healthy obese; MUN, metabolically unhealthy nonobese; MUO, metabolically unhealthy obese.

Next, we investigated the association between metabolic phenotypes and MACE. Over a median follow-up of 4.5 years (IQR: 3.8-5.9), 392 (1.7%) MACE occurred. Cumulative incidence curves for MACE (Fig. 3A) showed significant differences across phenotypes (log-rank *P* < .001). In univariable analyses, MUN and MUO phenotypes were associated with increased risks for MACE compared to the reference metabolic phenotype MHN (MUN: HR = 2.20 [95% CI: 1.66-2.92], *P* < .001; MUO: HR = 2.34 [95% CI: 1.54-3.55], *P* < .001, Fig. 3A).

After adjustment for age, sex, and smoking status, these associations remained significant (MUN: adjusted HR = 1.55 [95% CI: 1.16-2.07], *P* = .003; MUO: adjusted HR = 1.95 [95% CI: 1.28-2.97], *P* = .002, Fig. 3A). By contrast, there was no significant association between the MHO phenotype and MACE compared to the MHN reference phenotype (adjusted HR = 1.23 [95% CI, 0.89-1.70], *P* = .201).

Finally, we explored the association between metabolic phenotypes and all-cause mortality.

Over a median follow-up of 5.1 years (IQR: 4.3-6.5), 396 (1.7%) all-cause deaths occurred. Kaplan-Meier survival curves for all-cause mortality (Fig. 3B) showed significant differences across phenotypes (log-rank *P* < .001). In univariable analyses, all phenotypes compared to the reference phenotype MHN were associated with increased risks for all-cause mortality (MHO: HR = 1.45 [95% CI: 1.08-1.93], *P* = .013; MUN: HR = 2.13 [95% CI: 1.59-2.85], *P* < .001; MUO: HR = 2.59 [95% CI: 1.73-3.88], *P* < .001, Fig. 3B). After adjustment for age, sex, and smoking status, these associations remained significant (MHO: adjusted HR = 1.65 [95% CI: 1.23-2.21], *P* < .001; MUN: adjusted HR = 1.41 [95% CI: 1.05-1.90], *P* = .021; MUO: adjusted HR = 2.10 [95% CI: 1.41-3.15], *P* < .001, Fig. 3B).

Sensitivity analyses using the alternative metabolic health definition showed consistent associations for both MACE [Table S3 (21), (Zenodo)] and all-cause mortality [Table S4 (21), (Zenodo)].

## Discussion

In this study, we investigated the associations of metabolic phenotypes with CMR parameters and clinical outcomes in the UKB population. Our main findings were (1) different patterns of cardiac adaptation were present in distinct metabolic phenotypes, with increased LVEDV (MHO: +4.67 mL/m, MUO: +5.14 mL/m) and CO (MHO: +0.30 L/min/m, MUO: +0.31 L/min/m) in both obesity phenotypes, whereas MUN showed reduced LVEDV (−1.25 mL/m) compared to the MHN reference phenotype. LVEF was increased in MUN (+0.29%) and decreased in MUO (−0.48%). Additionally, WT was elevated across all phenotypes (MHO: +0.53 mm, MUN: +0.24 mm, MUO: +0.66 mm), and LVM/LVEDV ratio was increased in all groups (MHO: +0.06 g/mL, MUN: +0.04 g/mL, MUO: +0.08 g/mL); and (2) MUN and MUO phenotypes showed a significantly increased risk of MACE compared to the MHN reference phenotype (55% and 95% increase, respectively), while all 3 phenotypes showed an increased risk of all-cause mortality (65%, 41%, and 110% increase, respectively), with MUO showing the highest risk.

Metabolic homeostasis is paramount to cardiovascular health. MetS and its components are independent risk factors for mortality from CVD and all-cause death (4, 22). Accordingly, large-scale population-based studies have demonstrated increased CVD and mortality risks among MUN and MUO individuals (23, 24). Concordant with these results, we found significantly increased risks for both MACE and all-cause mortality in metabolically unhealthy phenotypes, with MUO individuals showing the highest risk. These findings align well with recent data from a UKB study by Zhou et al, which showed that rates of adverse cardiovascular and respiratory outcomes were highest in MUO individuals, followed by MUN (8). It is important to note that the metabolic health definition of this study was based on laboratory parameters, whereas our assessment relied on clinical diagnoses for hypertension and diabetes. The optimal definition of metabolic health remains debated, with various studies using different combinations of MetS components (7, 25, 26). Our sensitivity analysis using a more inclusive definition—classifying participants as metabolically unhealthy with any single MetS component—resulted in substantial reclassification yet highly consistent HRs, underscoring the robustness of our findings.

In contrast to our findings, data from the PROMISE study (which enrolled patients with chest pain but without a history of CVD) and the EUROPA study (which included patients with established cardiovascular disease) demonstrated lower CVD event risks for MUO compared to MUN (7, 27). Including patients with manifest CVD, these studies might have systematically excluded the highest-risk MUO individuals who experience premature mortality. MUO patients who do enter such studies may represent a relatively healthier subset with fewer comorbidities, potentially explaining their unexpectedly lower event rates compared to MUN individuals. The higher burden of comorbidities in MUO patients in our cohort likely contributes to their elevated mortality risk compared to MUN (8).

Our study extends prior work by combining comprehensive CMR characterization of metabolic phenotypes with prospective outcome assessment. While previous studies have separately examined either metabolic phenotype outcomes or cardiac structure in cross-sectional analyses, our study uniquely integrates both approaches in a large population-based cohort (7, 8, 23, 24, 28). Unlike studies in selected populations with prevalent cardiovascular disease, our population-based approach captures the spectrum of cardiac characteristics across metabolic phenotypes before clinical disease manifests (7, 27).

While obesity is linked to increased mortality, there is conflicting evidence as to whether the coexistence of MetS is the primary driver of this association (29, 30). Several studies have reported that obesity is associated with adverse CVD outcomes independent of metabolic health status (5, 31, 32). However, others found a paradoxical protective effect of obesity in heart failure, coronary artery disease, and cancer, termed the “obesity paradox” (33-35). Potential explanations for this phenomenon include a lack of cachexia, higher muscle content, and increased metabolic reserves (36-38). Our results extend these findings, as the MHO phenotype did not show an increased risk for MACE compared to the MHN reference phenotype. However, there is growing evidence suggesting methodological explanations rather than a true mechanistic relationship, such as misclassification bias caused by using BMI as the sole measure of obesity (39). Importantly, our observation that the MHO phenotype demonstrated an increased risk for all-cause mortality further challenges the concept of “healthy obesity.” The finding that MHO was associated with increased all-cause mortality but not MACE suggests that excess mortality may be driven by noncardiovascular mechanisms, including obesity-related malignancies, respiratory complications, chronic kidney disease, and hepatobiliary diseases (40-43). Additionally, our MACE composite primarily captures atherosclerotic events and may not fully reflect other cardiovascular complications, such as heart failure with preserved ejection fraction or arrhythmias, which are more prevalent in obesity (44).

The CMR results demonstrate distinct patterns in cardiac structure and function across metabolic phenotypes. In obesity, increased total blood volume and CO are necessary to meet heightened metabolic demands (45). Consistent with this physiology, both MHO and MUO individuals exhibited increased LVEDV and CO, with MUO displaying reduced LVEF (46). In contrast, MUN individuals displayed reduced LVEDV but increased CO with preserved LVEF, a hemodynamic profile consistent with lean hypertension, where enhanced sympathetic activity and increased peripheral vascular resistance drive CO through augmented heart rate rather than volume expansion (47, 48).

Analysis of cardiac remodeling revealed modest increases in LVM in metabolically unhealthy phenotypes, while MHO showed no significant elevations. All 3 phenotypes exhibited increases in WT and LVM/LVEDV ratio, reflecting geometric changes trending toward concentricity, though values remained largely within normal ranges (49). Notably, LAV was selectively elevated in metabolically unhealthy phenotypes but not MHO, suggesting that metabolic dysfunction, rather than obesity per se, may drive early diastolic impairment and increased filling pressures (50).

Collectively, these findings indicate a graded relationship between metabolic dysfunction and cardiac adaptation: obesity alone produces primarily volume-related changes, whereas metabolic abnormalities drive both geometric remodeling and diastolic impairment. The subtle cardiac adaptations observed across phenotypes, while largely within normal limits, may represent early compensatory mechanisms that precede overt structural disease. The association with adverse outcomes despite relatively preserved cardiac parameters suggests that conventional CMR measures may incompletely capture relevant pathophysiological changes. Future research incorporating advanced imaging techniques such as T1 mapping may help detect tissue-level abnormalities, including diffuse myocardial fibrosis, that could explain the increased cardiovascular risk in these phenotypes (51).

Our study has several limitations. First, although our sample size is large, the UKB cohort is not representative of the general population and includes a limited age range, which may limit generalizability (52). Second, our classification of metabolic health was based on laboratory parameters taken at the baseline assessment and clinical diagnoses for hypertension and diabetes prior to imaging, which may introduce differential misclassification bias for metabolically unhealthy phenotypes. Third, kidney function measurements were not available, limiting our ability to assess the role of renal dysfunction as a potential mechanistic pathway linking metabolic phenotypes to adverse outcomes (53). Fourth, our analyses lack longitudinal CMR data, which limits the ability to assess how changes in CMR parameters progress over time. Therefore, future studies with serial imaging in diverse populations are needed to better understand the temporal relationship between obesity, metabolic status, and cardiac health.

## Conclusions

In conclusion, our prospective analysis demonstrates that metabolic phenotype classification provides important prognostic information for cardiovascular events and mortality beyond traditional risk factors. Our CMR findings reveal that distinct patterns of cardiac structure and function across metabolic phenotypes are present years before clinical events manifest. These findings support integrating both metabolic health status and obesity into comprehensive cardiovascular risk assessment strategies. Future investigations should examine whether early cardiac changes detected by CMR can guide personalized prevention strategies to improve outcomes across metabolic phenotypes.

## Data Availability

Restrictions apply to the availability of some or all data generated or analyzed during this study to preserve patient confidentiality or because they were used under license. The corresponding author will on request detail the restrictions and any conditions under which access to some data may be provided.

## Abbreviations

BMI: body mass index
BSA: body surface area
CI: confidence interval
CMR: cardiac magnetic resonance imaging
CO: cardiac output
CVD: cardiovascular disease
HDL: high-density lipoprotein
HR: hazard ratio
ICD-9: International Classification of Diseases, Ninth Revision
ICD-10: International Classification of Diseases, Tenth Revision
IQR: interquartile range
LAV: left atrial volume
LVEDV: left ventricular end-diastolic volume
LVEF: left ventricular ejection fraction
LVM: left ventricular mass
MACE: major adverse cardiovascular events
MetS: metabolic syndrome
MHN: metabolically healthy nonobese
MHO: metabolically healthy obese
MUN: metabolically unhealthy nonobese
MUO: metabolically unhealthy obese
UKB: UK Biobank
WT: global left ventricular mean wall thickness
